# CB2 Cannabinoid Receptor as a Potential Target in Myocardial Infarction: Exploration of Molecular Pathogenesis and Therapeutic Strategies

**DOI:** 10.3390/ijms25031683

**Published:** 2024-01-30

**Authors:** Sagar A. More, Rucha S. Deore, Harshal D. Pawar, Charu Sharma, Kartik T. Nakhate, Sumit S. Rathod, Shreesh Ojha, Sameer N. Goyal

**Affiliations:** 1 Department of Pharmacology, Shri Vile Parle Kelavani Mandal’s Institute of Pharmacy, Dhule 424001, Maharashtra, India; sagarmore9424@gmail.com (S.A.M.); ruchadeore2012@gmail.com (R.S.D.); harshal.pawar@svkm.ac.in (H.D.P.); kartik.nakhate@svkm.ac.in (K.T.N.); sumit.rathod@svkm.ac.in (S.S.R.); 2 Department of Internal Medicine, College of Medicine and Health Sciences, United Arab Emirates University, Al Ain P.O. Box 15551, United Arab Emirates; charusharma@uaeu.ac.ae; 3 Department of Pharmacology and Therapeutics, College of Medicine and Health Sciences, United Arab Emirates University, Al Ain P.O. Box 15551, United Arab Emirates

**Keywords:** CB2 receptors, myocardial infarction, ROS, cytokines, cardiac markers

## Abstract

The lipid endocannabinoid system has recently emerged as a novel therapeutic target for several inflammatory and tissue-damaging diseases, including those affecting the cardiovascular system. The primary targets of cannabinoids are cannabinoid type 1 (CB1) and 2 (CB2) receptors. The CB2 receptor is expressed in the cardiomyocytes. While the pathological changes in the myocardium upregulate the CB2 receptor, genetic deletion of the receptor aggravates the changes. The CB2 receptor plays a crucial role in attenuating the advancement of myocardial infarction (MI)-associated pathological changes in the myocardium. Activation of CB2 receptors exerts cardioprotection in MI via numerous molecular pathways. For instance, delta-9-tetrahydrocannabinol attenuated the progression of MI via modulation of the CB2 receptor-dependent anti-inflammatory mechanisms, including suppression of pro-inflammatory cytokines like IL-6, TNF-α, and IL-1β. Through similar mechanisms, natural and synthetic CB2 receptor ligands repair myocardial tissue damage. This review aims to offer an in-depth discussion on the ameliorative potential of CB2 receptors in myocardial injuries induced by a variety of pathogenic mechanisms. Further, the modulation of autophagy, TGF-β/Smad3 signaling, MPTP opening, and ROS production are discussed. The molecular correlation of CB2 receptors with cardiac injury markers, such as troponin I, LDH1, and CK-MB, is explored. Special attention has been paid to novel insights into the potential therapeutic implications of CB2 receptor activation in MI.

## 1. Introduction

Myocardial infarction (MI) is one of the leading causes of mortality worldwide. The damage to a specific portion of the myocardium due to prolonged ischemia in MI eventually manifests as decline in the systolic and diastolic functions, and increases the risk of arrhythmias [1]. Despite the availability of several drugs, the mortality rate associated with acute MI is still high. While most deaths occur prior to reaching the hospital, nearly 10% of survivors die and about 50% require rehospitalization in the first 12 months post-MI [2]. Moreover, survivors remain at a higher risk of death for no less than 7 years following the first or recurrent MI episode [3]. Therefore, the underlying pathological mechanisms and novel molecular targets for MI need to be explored further to develop more effective therapies, minimize post-MI complications, and improve the survival. Coronary atherosclerosis developed due to conditions like obesity, diabetes mellitus, hypertension, and dyslipidemia, is the main pathogenic cause of MI [4,5]. In the majority of cases of acute MI, the rupture of atherosclerotic plaque occurs, which consequently leads to thrombus formation. A higher level of low-density lipoprotein (LDL) cholesterol is the key initiating factor for the development of atherosclerosis [6]. The oxidation and glycation of accumulated LDL cholesterol triggers the pro-inflammatory pathways, which contribute to atherosclerotic plaque development in the coronary artery [7]. Pro-inflammatory cytokines, such as tumor necrosis factor-alpha (TNF-α), interleukin-6 (IL-6), and interleukin-1β (IL-1β), are elevated in MI [8]. Normal endothelium expresses nitric oxide synthase (NOS) in response to stimuli, such as shear stress [9]; however, oxidized LDL cholesterol depletes nitric oxide (NO) production, which contributes to impaired endothelium-dependent vasodilation, and enhanced aggregation of platelets and leukocytes [10,11]. Atherosclerotic endothelium leads to the vasoconstrictive and prothrombotic environment that increases occlusive thrombus formation during plaque rupture [6,12]. However, inducible NOS (iNOS) present in atherosclerotic plaque may contribute to the inflammatory process, as a result of increased peroxynitrite and lipid hydroperoxide levels [13]. Oxidative stress in cardiomyocytes, characterized by higher levels of reactive oxygen species (ROS), is linked to the accumulation of harmful oxidants like malonaldehyde (MDA) and a reduction in protective antioxidants like reduced form of glutathione (GSH), leading to myocardial damage [14]. The modulation of the mitochondrial permeability transition pore (MPTP) is associated with electron transport chain-derived ROS, which influences cellular mitochondrial function in the myocardium [15]. MI is diagnosed by abnormalities in ECG waves and the detection of several biochemical markers of myocardial injury in the blood samples of MI patients [16,17].

Several natural and synthetic cannabinoids, as well as non-cannabinoid agents, have been explored for their ameliorative effects in regard to MI. Available pieces of evidence suggest that the endocannabinoid system plays a crucial role in the pathogenesis of MI [18,19,20]. By regulating the movement of myeloid cells, the changes in endocannabinoid cascades have been shown to support cardiac repair and functioning in MI [18]. The primary targets of cannabinoids are cannabinoid type 1 (CB1) and 2 (CB2) receptors, which belong to the G-protein-coupled receptor family [21]. While both receptors are present in the central nervous system [22,23], CB2 receptors are also abundant in the periphery, including the heart [24], liver, and immune cells [25,26,27,28,29]. It has been suggested that the CB2 receptor modulates the development of a variety of immunological and inflammatory illnesses, including atherosclerosis and ischemic heart disease [30,31,32,33,34]. Further, the activation of CB2 receptors in cardiomyocytes alleviates several pathological changes associated with myocardial injury, by modulating several downstream molecular pathways [35,36,37,38,39].

This review intends to provide a comprehensive analysis of the molecular pathways activated by CB2 receptors in the context of MI. These pathways incorporate various aspects, including autophagy induction, the regulation of ROS (oxidant and antioxidant mechanism), poly (ADP-ribose) polymerase-1 (PARP-1) activity, influence on the MPTP opening, and their relevance in both experimental and clinical studies. Furthermore, we discuss the molecular associations between CB2 receptors and cardiac injury indicators, such as troponin I, lactate dehydrogenase (LDH), and creatine kinase MB (CK-MB). Simultaneously, the correlation between CB2 receptors and pro-inflammatory cytokines, such as TNF-α, IL-6, and IL-1β in MI is discussed. Particular emphasis has been placed on uncovering new perspectives regarding the potential therapeutic benefits of activating CB2 receptors in the context of MI. 

## 2. CB2 Receptor in the Cardiovascular System

As far as discovery is concerned, the human leukemic cell line HL-60 was first utilized to clone the CB2 receptor in 1993 [40]. Later studies suggest that these receptors are abundantly present in the spleen and immune cells, and are moderately expressed in several peripheral tissues, including cardiovascular tissues [24,41]. In MI, damage occurs to the cardiomyocytes in the ischemic zone, accompanied by the formation of a fibrotic scar [42]. It is interesting to note that the presence of CB2 receptors in the myocardium and endothelium of the larger arteries might be responsible for cardioprotection against ischemia [43,44,45]. Recent data suggest that CB2 receptors protect the heart against MI when the diabetic condition is involved. Activation of CB2 receptors in diabetic mice with MI compensated with hemodynamic fluctuations and protected the myocardium, which was restored by the enzymatic markers of the injury, and decreased the oxidative stress and cytokines levels [38]. In diabetic animals, the pharmacological activation of CB2 receptors exerts a cardioprotective effect by restoring the cardiac equilibrium of iNOS/eNOS [46], and attenuating oxidative stress, inflammation, fibrosis, and cell death [47], whereas the aggravation of myocardial pathology was associated with the genetic deletion of CB2 receptors [47]. In addition, the expression of myocardial CB2 receptors increased in response to pathophysiological events like chronic heart failure [48], ischemic cardiomyopathy [49], and severe systemic inflammation [39], playing a key role in cardioprotection. CB2 receptor upregulation was observed in the heart of mice with hepatic cardiomyopathy, and the activation of this receptor markedly improved myocardial inflammation and cardiac dysfunction [50]. CB2 receptor activation by a natural cannabinoid, delta-9-tetrahydrocannabinol (Δ-9-THC), protects cardiomyocytes from hypoxia [51] and ischemia–reperfusion-induced injury [52].

In healthy state, the vascular endothelium acts as a highly permeable barrier with anti-adhesive properties, isolating the blood vessel wall from surrounding tissues [53]. Endothelial injury leads to increased permeability, sub-endothelial lipid buildup, the upregulation of adhesion molecules, the release of cytokines and growth factors, as well as platelet and monocyte adherence to it [54]. These cells can regulate inflammation, hemostasis, angiogenesis, and vascular tone. As a result, endothelial dysfunction has a major impact on cardiovascular characteristics that may ultimately result in MI [55,56]. Pro-inflammatory mediators and monocyte migration worsen endothelial damage. Interestingly, CB2 receptors protect endothelial cells in response to pro-inflammatory cytokine TNF-α [57]. Also, CB2 receptor activation decreases the subendothelial accumulation of oxidized LDL by suppressing the production of pro-inflammatory cytokines like IL-10, IL-12, and TNFα [58]. NO is crucial for preconditioning and other cardio-protective mechanisms. The activation of CB2 receptors with Δ-9-THC increases NO production to induce vasodilation [51].

## 3. Functional Differences and Rivalry between the CB2 Receptor and the CB1 Receptor in Myocardial Injury

Although CB1 and CB2 receptors are expressed in the cardiovascular tissues of rodents [44,50] and humans [24,48,59], both show functional differences in a variety of myocardial injuries. The first study on the cardioprotective functions of these receptors showed that blocking of the CB2 receptor with an antagonist SR144528 diminished the cardioprotective action of lipopolysaccharide against ischemic injury, while the CB1 receptor antagonist SR141716A had no effect [60]. Similarly, CB2 receptor blockade, but not the CB1 receptor, causes the reversal of cardioprotection by heat stress-mediated preconditioning against ischemia–reperfusion-induced injury in an isolated heart [61]. The cardioprotective effects of both 2-arachidonoylglycerol (endogenous cannabinoid) and palmitoylethanolamide (endocannabinoid-like lipid mediator) were abolished completely by the CB2 receptor antagonist, whereas CB1 receptor antagonism can partially block the effect of 2-arachidonoylglycerol only [62]. In CB2 receptor knock-out hearts, the CB2 receptor agonist JWH133 failed to protect the myocardium against ischemic injury. The results further suggest that CB2 receptor activation may protect against post-ischemia–reperfusion heart failure through the direct inhibition of cardiac myocyte and fibroblast death and prevention of myofibroblast activation [63]. These variations were attributed to the difference in the expression of CB1 and CB2 receptors in cardiac tissue. A comparative evaluation of the protein and mRNA for both receptors indicates the localization of the CB1 receptor almost exclusively on arterial and capillary endothelial cells in intact hearts, while the CB2 receptor appeared in the cardiomyocytes and endothelial cells of larger arteries [44]. Therefore, NO produced by cardiac vascular endothelium plays an important role in the actions of CB1 and CB2 receptors in the heart. For instance, in isolated neonatal cardiomyocytes, the protective action of the phytocannabinoid, Δ-9-THC, against hypoxia was dependent on NO production, which was sensitive to CB2 receptor, but not CB1 receptor, antagonism. The CB2 receptor selective effect was observed, as neonatal heart cells express the CB2 receptor, but not the CB1 receptor [51]. However, in a mature heart, the NOS inhibitor N(G)-nitro-L-arginine attenuated the cardioprotective effect of the selective CB1 receptor agonist ACEA, while failing to prevent the cardioprotective action of the CB2 receptor agonist, JWH015, against ischemia–reperfusion-triggered myocardial injury. Since CB1 receptors are present mainly on endothelial cells in the heart, they exert their effect by producing NO. On the contrary, CB2 receptors are highly expressed in the cardiomyocytes and, therefore, exert a cardioprotective effect directly, independent of NO [44]. A similar NO-dependent cardioprotective effect of CB1 receptors and NO-independent action of the CB2 receptor was reported in another study [64].

While the above reports indicate ameliorative actions of both CB1 and CB2 receptors in myocardial injuries, comparative studies suggesting the cardiodamaging role of CB1 receptors and the cardioprotective function of CB2 receptors are also available. Myocardial inflammatory infiltration and fibrotic lesions induced by the chronic administration of the antipsychotic drug, clozapine, was blunted by selective CB1 receptor antagonists, AM281 and rimonabant, but not by the selective agonist arachidonyl-2′-chloroethylamide. In contrast, selective CB2 receptor agonists, JWH133 and AM1241, but not the selective antagonist AM630, blunted clozapine-triggered cardiotoxicity [65]. Similar responses by CB1 and CB2 receptor modulators were observed on the cardiotoxicity induced by another antipsychotic agent, quetiapine [66]. Pharmacological activation of CB2 receptors protects against ethanol-induced myocardial damage [67]. However, in the case of doxorubicin-induced cardiotoxicity, pharmacological inhibition of CB1 receptors, but not the activation or inhibition of CB2 receptors, produced ameliorative action on cardiomyocytes [68]. MI in the diabetic condition usually appears more extensive and severe. In diabetic animals, the pharmacological inhibition or genetic deletion of CB1 receptors [69] and activation of CB2 receptors exert a cardioprotective effect [46], whereas the aggravation of myocardial pathology was associated with the genetic deletion of CB2 receptors [47]. Myocardial hypertrophy significantly increases the risk of MI. While CB2 receptor deficiency is associated with cardiac hypertrophy [70], CB1 receptor antagonism attenuates hypertrophic changes in the heart [71]. Myocardial fibrosis following MI facilitates the development of cardiac dysfunction and arrhythmias. The inhibition of CB1 receptors [72] and activation of CB2 receptors [28] retarded cardiac fibrosis following MI and improved the cardiac function. In patients with severe heart failure, CB1 mRNA expression was downregulated 0.7-fold, while 11-fold upregulation in CB2 receptor expression was observed [48].

Viewed collectively, the data suggest that CB1 receptors aggravate myocardial injury, although the reports are inconsistent. On the other hand, CB2 receptors clearly offer cardioprotection in a variety of conditions. Further, the availability of a high density of CB2 receptors on cardiomyocytes can offer protection against ischemic injury by modulating downstream molecular pathways.

## 4. CB2 Receptor-Dependent Molecular Mechanisms in MI

The available literature suggests several CB2 receptor-dependent molecular mechanisms that are responsible for cardioprotection in MI.

### 4.1. CB2 Receptor Protects against MI through the Induction of Autophagy

The degradation of damaged proteins and organelles into amino acids and fatty acids for energy production and their recycling is accomplished by the metabolic process of autophagy [73,74]. Moreover, autophagy is triggered in response to food deprivation or metabolic stress to maintain tissue function and homeostasis. For instance, autophagy plays an important role in protecting cardiomyocytes from ischemic stress or reperfusion injury [75]. Atherosclerosis, MI, ischemic stroke, and multiple sclerosis are modulated by autophagy [76,77,78]. By preventing the development and progression of MI, autophagy has been shown to have a protective impact on the heart [79]. According to earlier research, when the CB2 receptor was selectively activated with HU308, it had a cardioprotective impact over diabetic cardiomyopathy and shielded the cardiomyocytes from the damaging effects of high glucose by activating autophagy through the AMPK–mTOR–p70S6K signaling pathway [80]. Furthermore, in several kinds of cellular and animal models, CB2 receptor activation has been demonstrated to enhance autophagy [81]. CB2 receptor activation-induced bone marrow differentiation in vitro is associated with the induction of autophagy and p62-linked Nrf2 depletion [82]. Animals were protected against cardiac ischemia–reperfusion injury by the synthetic CB2 receptor agonist AM1241, which activates the Pink1/Parkin pathway to initiate autophagy [83]. In addition, scientists examined the amounts of autophagy-related proteins such as Beclin-1, LC3-II/I, and p62 to ascertain the relationship between the CB2 receptor and autophagy in MI. Deletion of the CB2 receptor increases the quantity of p62 and decreases Beclin-1 and LC3-II/I ratio levels, suggesting that the CB2 receptor has a beneficial influence on autophagy in MI [84].

**Figure 1:**
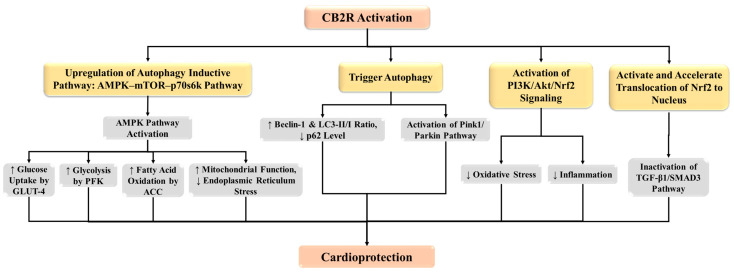
CB2 receptor protects the myocardium via induction of autophagy, and activation of PI3K/Akt/Nrf2 signaling. ACC: acetyl-CoA carboxylases; Akt: protein kinase; AMPK: AMP-activated protein kinase; CB2R: CB2 receptor; GLUT-4: glucose transporter type 4; mTOR: mammalian target of rapamycin; Nrf2: nuclear factor erythroid 2-related factor 2; PFK: phosphofructokinase; p70S6K: 70-kDa ribosomal protein S6 kinase; PI3K: phosphoinositide 3-kinase; TGF-β1: transforming growth factor beta-1.

AMP-activated protein kinase (AMPK) activation is an essential component of the adaptive response to cardiomyocyte stress that occurs during myocardial ischemia. Pharmacological activation of AMPK prevents myocardial necrosis and contractile dysfunction during ischemia–reperfusion and potentially represents a cardioprotective strategy for the treatment of MI [85]. Interestingly, activation of AMPK signaling pathways by CB13, a non-selective CB1 and CB2 receptor agonist, produces antihypertrophic effects, and reduces mitochondrial dysfunction in cardiomyocytes [86,87]. While the AMPK–mTOR–p70S6K signaling pathway is considered a classic inductive pathway of autophagy, the CB2 receptor seems to regulate this pathway and plays a cardioprotective role in MI. In CB2 receptor knock-out animals, the downregulated AMPK–mTOR–p70S6K signaling results in impaired autophagy, which is accountable for the worsening of MI [84]. It is interesting to note that during ischemia–reperfusion, AMPK activation enhances glucose uptake by glucose transporter 4, fatty acid oxidation by acetyl-coenzyme A carboxylase, and glycolysis by phosphofructokinase [88], attenuates mitochondrial dysfunction [86], and decreases endoplasmic reticulum stress [89]. Therefore, the CB2 receptor can augment these changes via the activation of AMPK pathways to offer cardioprotection (Figure 1).

### 4.2. CB2 Receptor Protects against Myocardial Fibrosis in MI via Modulation of Transforming Growth Factor Beta (TGF-β)/Small Mother against Decapentaplegic Homolog 3 (Smad3) Superfamilies Signaling Pathway

As one of the main reasons for inadequate remodeling in heart failure following MI, cardiac fibrosis must be prevented and reversed to effectively treat cardiovascular complications [90]. Growing research on the architectural domains of non-coding RNAs indicates that different miRNAs play significant roles in cardiac fibrosis following MI by controlling the TGF-β/Smad3 signaling cascade. Infarcted myocardial and hypoxia-associated cardiac fibroblasts downregulate miR-130a, and overexpression of this protein can shrink the infarcted area and lessen the severity of cardiac impairment. This mechanism of action might be associated with the modulation of TGF-β/Smad3 signaling activity and the suppression of cardiac fibroblasts’ transformation into myofibroblasts by the immediate inhibition of TGF-βR1 [91]. Comparably, the suppression of miR-328 prevented cardiac fibrosis in mice following MI, while the overexpression of miR-328 was seen in the hearts and cardiac fibroblasts of MI animals. Additionally, TGF-βRIII is the primary target of miR-328, and the overexpression of miR-328 can target and block TGF-βRIII, which in turn activates the TGF-β1 signal pathway and stimulates the synthesis of collagen [92]. It has long been known that TGF-β1 is essential for maintaining tissue homeostasis and regulating the extracellular matrix. Cell signaling is mostly transmitted by TGF-β1 via the downstream mediator protein Smad3. The TGF-β1/Smad3 pathway is a classic mechanism in the progression of organ fibrosis, as evidenced by several studies [93,94,95].

Previous research indicates that CB2 receptor activation prevents fibrosis in several organs. The CB2 receptor agonist has been shown to significantly lower the amount of collagen in the liver in cirrhotic rats and to have antifibrotic effects in mice with experimental dermal fibrosis [96,97]. Furthermore, in several types of disease models, CB2 activation has demonstrated significant potential in reducing oxidative stress and inflammation [98,99,100]. In earlier research, the CB2 receptor agonist AM1241, which inhibits increased oxidative stress and inflammation in ischemic hearts, activates PI3K/Akt/Nrf2 transmission to support endogenous myocardial regeneration [36]. It is a well-known fact that increased levels of oxidative stress and inflammation in the heart exacerbate cardiac fibrosis [101,102]. The available evidence shows that CB2 receptor activation may activate the PI3K/Akt pathway [36,103]. It has also been shown that Nrf2, a well-known anti-oxidative protein that is commonly linked to antifibrotic effects, could be activated by activating Akt [104,105]. Previous studies have shown that in myocardial infarcted mice, the CB2 receptor agonist AM1241, improved cardiac functional recovery and reduced cardiac fibrosis. AM1241, in cardiac fibroblasts injured by H/SD, prevented the transition of cardiac fibroblasts into myoblasts and the synthesis of collagen, including collagen I and collagen III, in a way that was dependent on Nrf2. Furthermore, AM1241 increases superoxide dismutase (SOD) and GSH levels and decreases MDA formation and ROS generation. In addition, AM1241 stimulated and sped up Nrf2’s translocation to the nucleus and, in an Nrf2-dependent manner, blocked the TGF-β1/Smad3 pathway [28]. Based on these findings, we hypothesized that the protective role of the CB2 receptor against myocardial fibrosis may be associated with the modulation of the Nrf2–TGβ1–Smad3 complex pathway in cardiomyocytes (Figure 1).

### 4.3. CB2 Receptor Suppresses Poly (ADP-Ribose) Polymerase-1 (PARP-1) Activity in MI

The most prevalent isoform of the PARP enzyme family, which is constantly expanding, is the nuclear enzyme PARP-1 [106,107]. Multiple physiological and pathophysiological cellular functions, including the repair of DNA, the transcription of genes, cell cycle progression, cell death, the chromatin function, and genomic stability are regulated by poly ADP-ribosylation [106,107,108,109]. PARP-1 is involved in the transcriptional regulation of multiple proteins involved in inflammation, such as cyclooxygenase-2, intercellular adhesion molecule-1 (ICAM-1), and iNOS. This regulation of protein expression is particularly significant. In vitro models of inflammation, circulatory shock, and ischemia–reperfusion showed that the lack of functional PARP-1 (genetic or pharmacological) also decreased tissue infiltration with activated phagocytes, and the expression of a variety of pro-inflammatory mediators, such as cytokines, chemokines, adhesion molecules, and enzymes [109]. It has been demonstrated that PARP functions as a co-activator in NF–κB-mediated transcription, which is a crucial transcription factor in controlling the expression of these proteins [110]. Poly ADP-ribosylation can relax chromatin structure, increasing the accessibility of genes for the mechanism of transcription. These groundbreaking findings have been expanded upon to demonstrate the involvement of PARP-1 in the activation of other crucial pro-inflammatory signaling cascades, including p38 MAPK [111] and JNK [112]. In a variety of cardiovascular disorders linked to acute (such as MI, coronary bypass, aortic reconstructive surgeries, and septic shock) and/or chronic inflammation (e.g., atherosclerosis and cardiovascular aging), PARP inhibitors can suppress the expression of pro-inflammatory genes.

**Figure 2:**
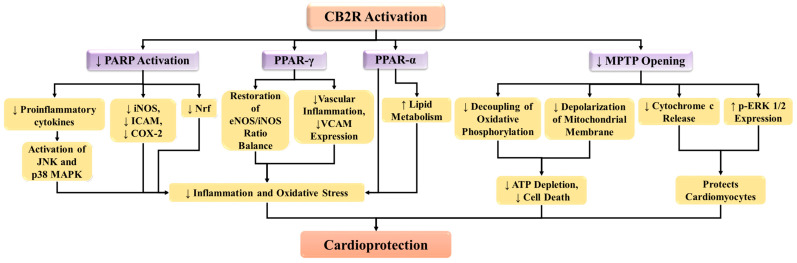
CB2 receptors protect cardiomyocytes via inhibition of PARP activation and MPTP opening, and possible activation of PPARs. CB2R: CB2 receptor; COX-2: cyclooxygenase-2; eNOS/iNOS: endothelial nitric oxide synthase, and inducible nitric oxide synthase; ICAM: intercellular adhesion molecule; iNOS: inducible nitric oxide synthase; JNK: c-Jun N-terminal kinase; MAPK: mitogen-activated protein kinase; MPTP: mitochondrial permeability transition pore; PARP: poly (ADP-ribose) polymerases; PPAR-α: peroxisome proliferator-activated receptor alpha; PPAR-γ: peroxisome proliferator-activated receptor gamma; VCAM: vascular cell adhesion molecule.

Preclinical studies have shown that treating diabetic mice with a CB2 receptor agonist JWH133 reduces elevated caspase and PARP activities in the myocardial tissue, along with chromatin fragmentation [47]. Moreover, JWH133 prevented the infiltration of inflammatory cells into serum and tissue TNF-α, macrophage inflammatory protein (MIP)-1α/CCL3 and MIP-2/CXCL2 levels, and adhesion molecule ICAM-1 expression in a mouse model of liver ischemia–reperfusion injury [113]. JWH133 has demonstrated the ability to reduce TNF-α-induced production of VCAM-1 and ICAM-1 in vitro [114]. Cannabinoids have been shown to play a protective function against ischemia–reperfusion damage in the heart, brain, and liver [99,100]. The key mechanism by which the cannabinoids’ protective impact is mediated is the triggering of the CB2 receptor, which inhibits the inflammatory response and reduces endothelial cell activity [115,116,117,118]. In preparations ranging from single myocytes and isolated hearts to the unaltered heart in vivo, endothelium-derived NO is produced physiologically across cardiac myocytes by an endothelial type of nitric oxide synthase (eNOS). It may regulate myocardial relaxation, diastolic tone, and oxygen consumption [119,120]. Additionally, the activation of CB2 receptors and the modulation of iNOS/eNOS cardiac equilibrium may be significant mechanisms for mediating the cardioprotective impact of the synthetic cannabinoid agonist, WIN55,212-2, towards ischemia–reperfusion injury in an experimental model of type 2 diabetes [46]. Similar to THC, WIN55,212-2, a dual CB1/CB2 agonist, has been demonstrated to have anti-atherosclerotic properties in rodents. Moreover, WIN55,212-2 inhibited the pro-inflammatory response and decreased activation of pro-inflammatory genes and NF-κB in the aortas of ApoE−/− mice [121] (Figure 2).

### 4.4. CB2 Receptor Modulates the Activity of Peroxisome Proliferator-Activated Receptors (PPAR)

PPARs are nuclear hormone receptor superfamily transcription factors that are ligand activated. There are three subtypes of PPARs: PPARα, PPARγ, and PPARβ/δ [122]. PPARα activation may protect the heart from ischemia–reperfusion injury by reducing oxidative stress and inflammation during blood flow restoration [123]. PPARα activation is believed to be cardioprotective due to its ability to enhance lipid metabolism, decrease inflammation, and increase overall heart health [124]. In contrast, mice with cardiac overexpression of PPARα displayed the worst recovery of cardiac power, the lowest rates of glucose oxidation, and the greatest levels of fatty acid oxidation. These results suggest a negative impact of persistent PPARα activation on cardiac recovery after ischemia [125]. In addition to PPARα, the cardioprotective actions of PPARγ are also recorded. PPARγ suppresses pathological changes associated with myocardial ischemia and reperfusion injury by mitigating oxidative stress, suppressing pro-inflammatory cytokines, improving the metabolism of glucose and lipids, and reversing apoptosis [126]. The crosstalk between PPARs and CB2 receptors is reported. A CB2 receptor agonist β-caryophyllene (BCP) decreased PPAR-γ expression in human articular chondrocytes stimulated with LPS. This was associated with its ameliorative effects in arthritis [127]. Furthermore, BCP was demonstrated to have anti-platelet aggregation in vitro [128], lower lipid content, and reduce oxidative damage in cardiac tissue [128,129]. BCP via CB2 receptors exerts its anti-inflammatory effects by inducing the PPAR-γ pathway [130,131].

PPAR-γ played a role in the reduction of vascular inflammation and VCAM-1 expression, as well as the restoration of a balanced eNOS/iNOS ratio by BCP, leading to the normalization of NO levels [132]. Therefore, it is possible that the CB2 receptor might produce its cardioprotective effects via its interaction with PPARα or PPARγ. This line of argument is supported by the fact that activation of CB2 receptors triggers PPARγ-dependent signaling to improve colitis [131]. As discussed above, although PPARα plays a pivotal role in protecting the heart during MI, its direct association with the CB2 receptor is not reported. Therefore, the possibility of CB2 receptor-mediated stimulation of PPARα, in addition to PPARγ activation, needs to be explored to establish its interaction in MI (Figure 2).

### 4.5. CB2 Receptor Inhibits Mitochondrial Permeability Transition Pore (MPTP) in MI

Acute ischemia–reperfusion injury is one of the main clinical symptoms of ischemic heart disease. This condition damages the heart and causes cardiac dysfunction, myocardial injury, and cardiomyocyte death, which can lead to cardiac arrhythmias, heart failure, and even death. The absence of oxygen in the injured region during MI prevents it from undergoing oxidative phosphorylation, and this energy deprivation causes the ions inside the myocyte to become dysregulated. The inner mitochondrial membrane has a non-selective pore called the MPTP, which may pass solutes up to 1.5 kDa in size [133]. The loss of the membrane potential of mitochondria, organelle enlargement, and ultimate rupture are caused by pore opening [134]. Further myocardial injury is caused after reperfusion, most likely through the entry point of the MPTP. Mitochondrial damage plays a crucial role in the loss of cardiomyocyte viability and function in reperfusion injury. Two major causes of mitochondrial malfunction are the generation of ROS and oxidative stress, brought on by the prolonged opening of MPTPs [135]. The opening of the MPTP causes depolarization of the mitochondrial membrane and decoupling of oxidative phosphorylation, which causes ATP depletion and cell death [136].

According to new research, the CB2 receptor can prevent the damage brought on by ischemia–reperfusion by exerting cardioprotective effects. The reduction of the infarct size upon development with CB2 receptor agonists prior to ischemia, or during reperfusion, in either ex vivo or in vivo preparations has demonstrated that CB2 receptors induce protective effects during the early steps of ischemia–reperfusion [137,138]. In isolated rat hearts subjected to low-flow ischemia and reperfusion, the cardiac restorative effect of endocannabinoids is eliminated by blocking CB2 receptors [62]. Acute CB2 receptor agonist injection before ischemia was shown to cushion the heart against ischemia–reperfusion damage, which was shown to enhance cardiac ventricular function recovery, increase coronary flow, and decrease the myocardial infarct size. Furthermore, it was also demonstrated that the CB2 receptor agonist, JWH133, exhibits significant cardioprotective activity by preventing MPTP opening to reduce the release of cytochrome c from the mitochondria and increases p-ERK1/2 expressions [139] (Figure 2).

## 5. CB2 Receptor Suppresses Oxidative Stress in MI

ROS include free radicals, such as superoxide anion (O_2_^−^), lipid radicals (ROO^−^), hydroxyl radical (HO^−^), NO, and non-free radicals species, such as hydrogen peroxide (H_2_O_2_), hypochlorous acid (HClO), and peroxynitrite (ONOO^−^), which have oxidizing effects and contribute to oxidative stress [140]. ROS are produced in the ischemic myocardium, especially after reperfusion, and contribute to myocardial necrosis [141,142]. The mitochondria, phagocytes, nicotinamide adenine dinucleotide phosphate (NADPH), nitrogen oxides (NOX), and xanthine oxidase are the major sources of ROS in the ischemic–reperfused myocardium [143,144].

Previous findings reported that CB2 receptor activation by HU308 could protect against ROS by inhibiting superoxide release in MI and the ischemia–reperfusion model [37]. In another study, similar results were observed for the CB2 receptor agonist, JWH133, during ischemia, significantly reducing oxidative stress in infarcted hearts after 24 h of reperfusion in a mouse model of MI [118]. Moreover, it has been confirmed in various animal models of atherosclerosis and myocardial ischemia–reperfusion injury that CB2 receptor activation by natural and synthetic agonists shows antioxidative effects [145]. Simultaneously, experimental data have also shown increased expression of NOX2 in cardiomyocytes after MI [146]. The increased expression of NOX2 was mainly observed in the infarcted area, although it was also present in cardiomyocytes away from the infarcted area [146]. A previous study reported that the activation of CB2 receptors by the administration of AM1261 attenuates angiotensin II-induced atrial fibrillation, by managing oxidative stress and the inflammatory response via a potential NOX/CaMKII mechanism [147].

Increased oxidative stress and decreased antioxidant defense play major roles in the pathogenesis of cardiovascular diseases [148]. GSH, SOD, and catalase protect cells against oxidative stress, and exert a protective role. GSH directly serves as a scavenger of electrophilic and oxidant species, or indirectly through enzymatic catalysis [149]. SOD converts superoxide radicals into H_2_O_2_, which is then reduced to water and oxygen by the catalase enzyme system [150]. It has been reported that GSH, SOD, and catalase levels are decreased in MI [151,152]. On the contrary, MDA is a biomarker of lipid peroxidation, which is often associated with cardiovascular disease. The measurement and follow-up of lipid peroxidation levels were proposed in a previous study for the diagnosis and treatment of acute coronary syndrome (ACS) patients [153]. Higher MDA levels were related to ischemic damage or unstable plaque in ACS patients [154], and can be correlated with acute MI [155]. Interestingly, CB2 receptor activation reduced oxidative stress and repaired antioxidant defense by elevating the levels of GSH, SOD, and catalase, and lowering the contents of MDA in isoproterenol-induced MI in diabetic mice [38]. These data suggest that CB2 receptor activation could protect against cardiac damage associated with ischemia by decreasing oxidative stress.

## 6. CB2 Receptor Suppresses Inflammatory Response in MI

The upregulation of pro-inflammatory mediators, like TNF-α, IL-1β and the family of IL-6, is responsible for the activation of leukocyte integrin, inducing endothelial cell adhesion and mediating the extensive adhesive interaction that ultimately leads to inflammatory cell extravasation into the myocardial infarct [156]. In experimental models of MI, there is a continuous and noticeable rise in the expression of pro-inflammatory cytokines. such as TNF-α, IL-1β, and IL-6 [157,158]. After MI, TNF-α is released, and it can lead to inflammatory damage by causing the infarcted myocardium to produce chemokine and adhesion molecules [159,160]. According to a previous finding, JWH133 reduced TNF-α-induced chemotaxis and integrin CD18/CD11b upregulation on human neutrophils in vitro, and suppressed oxidative stress and neutrophil infiltration in the infarcted myocardium in a mouse model of ischemia–reperfusion, suggesting a potential molecular explanation that could be relevant to human pathophysiology and helping to reduce the size of the infarct [118]. One more finding proved that the activation of the CB2 receptor significantly reduced the level of TNF-α in isoproterenol-induced MI in a diabetic mice model, which indicates that the CB2 receptor could protect the myocardium from the inflammatory response [38].

IL-1 is implicated in the pathophysiology of cardiac remodeling and is essential in inducing the post-infarction inflammatory response. For patients with MI, addressing the IL-1 signaling cascade may, therefore, be a promising therapeutic target [161]. Dead cardiomyocytes in a mouse model of MI release IL-1α [162] and, following infarction, IL-1β production is markedly increased [158]. CB2 receptor activation by curcumin significantly reduced the inflammatory response by depleting the synthesis of IL-1β in cardiomyocytes; on the other hand, the CB2 receptor blockade by AM630 significantly attenuated the effect of curcumin in isoproterenol-induced MI in diabetic mice, which indicates an anti-inflammatory effect of the CB2 receptor in cardiovascular diseases [38].

One of the first pleiotropic cytokines to be identified, IL-6, is involved in several physiological processes. The role of IL-6 in the development of cardiac insufficiency remains unclear, despite an abundance of experimental and clinical research. It is thought that elevated IL-6 levels may play a role in the initial stages of heart failure [163]. Treatment with curcumin showed a reduction in the levels of inflammatory mediators, such as IL-6, IL-1β, and TNF-α, in the myocardium in diabetic mice with myocardial infarction. This effect of curcumin was blocked by the CB2 receptor antagonist, AM630, indicating that curcumin exerts anti-inflammatory activity in the myocardium through activation of the CB2 receptor [38]. In the case of hepatic cardiomyopathy in mice, upregulation of the CB2 receptor was observed in the heart. The activation of CB2 receptors significantly reduced the serum TNF-α content, and improved oxidative stress, myocardial inflammation, and cardiac dysfunction [50]. A significant increase in CB2 receptor mRNA and protein was observed in murine embryonic cardiomyocytes in vitro, following cultivation under hypoxia or stimulation with pro-inflammatory cytokine interferon γ [164]. CB2 receptor-deficient mice with ischemia–reperfusion-triggered MI showed a cytokine-driven inflammatory response, which was linked with a non-compacted transmural scar, dysfunctional infarction border zone formation, and adverse myocardial remodeling. These changes are linked with the work by [165]. Overall, the reports suggest that CB2 receptor activation could protect the myocardium by suppressing inflammatory pathways.

## 7. CB2 Receptor and Cardiac Injury Markers

**Figure 3:**
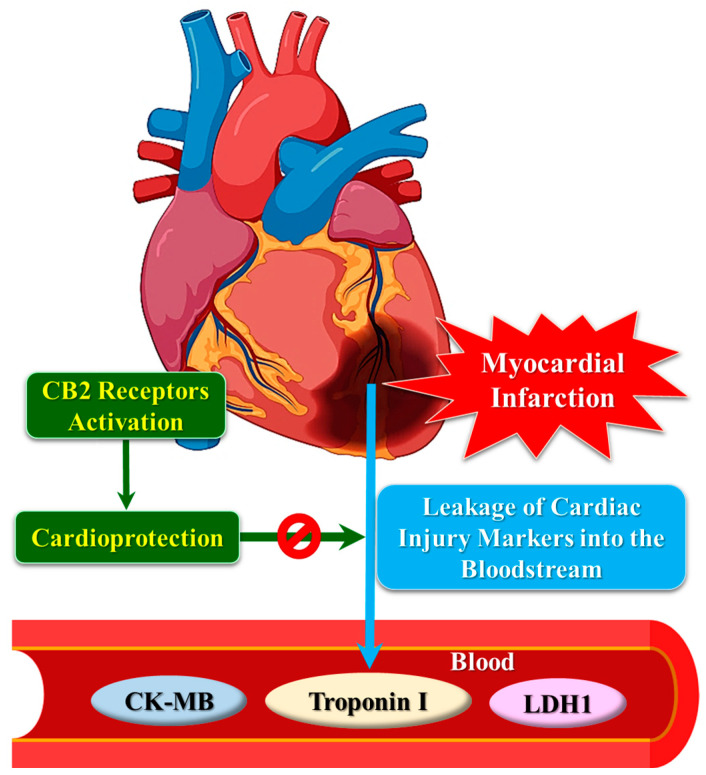
CB2 receptor activation attenuates the release of cardiac injury markers into the bloodstream, due to myocardial injury by exerting a cardioprotective effect. CK-MB: creatine kinase–myoglobin binding; LDH: lactate dehydrogenase.

Besides an ECG, the measurement of the levels of cardiac markers in serum is crucial for the diagnosis of MI [16,17]. These marker proteins are released into the bloodstream within a few hours after the onset of MI and remain elevated for several days. Since cannabinoids exhibit cardioprotective actions via the CB2 receptor, the altered expression of these receptors can consequently modulate the plasma levels of cardiac tissue injury markers. Interestingly, CB2 receptor activation restores the levels of some biomarkers following myocardial injury (Figure 3).

The influence of CB2 receptor activation on LDH has been studied extensively. Higher levels of LDH1 in the blood (LDH1:LDH2 ratio greater than one) are associated with acute MI [166]. Knocking out the CB2 receptor in primary myocardiocytes decreases the viability of cells and increases the release of LDH under oxygen–glucose deprivation [84]. In hypoxic conditions, the activation of CB2 receptors by Δ-9-THC induces iNOS to increase NO formation and protects cardiomyocytes from injury. This consequently prevents hypoxia-induced LDH leakage [51]. Moreover, Δ-9-THC also protects the myocardium against ischemia–reperfusion injury, confirmed by the increase in creatine kinase, total antioxidant capacity, and LDH in the myocardial tissue [52]. Activation of the CB2 receptor exerts a cardioprotective action in diabetic cardiomyopathy via the induction of AMPK–mTOR–p70S6K signaling-mediated autophagy and reduces LDH release [80]. In diabetic mice with myocardial fibrosis and hypertrophy, LDH was dramatically increased in the blood. BCP, a selective CB2 receptor agonist, attenuated these pathological changes in the myocardial tissue and reduced serum LDH significantly [167]. Similarly, curcumin was found to protect diabetic mice from cardiac damage via the CB2 receptor, indicated by restored LDH content in the myocardial tissue [38]. The involvement of the CB2 receptor was assumed based on the attenuation of the cardioprotective effects of curcumin by the CB2 receptor antagonist AM630. This was further supported by a molecular docking study, which revealed curcumin as a potent CB2 receptor agonist [38]. Molecular docking is widely employed alone, or jointly, with in vivo experiments to authenticate ligand–receptor interactions [168,169,170].

In mice with cecal ligation and puncture (CLP)-induced sepsis, increased expression of the CB2 receptor was observed. Interestingly, activation of the CB2 receptor by HU308 in these septic animals reduces the content of cardiac injury mediators, such as LDH, creatinine kinase–myocardial binding (CK-MB), IL-1β, and NLRP3 inflammasome, and activates caspase-1 and gasdermin D. The CB2 receptor-dependent changes were linked to the inhibition of CLP-triggered inflammatory programmed cell death (pyroptosis) in the myocardium [39]. Similar to HU308, which activates the CB2 receptor in septic animals to reduce the plasma content of CK-MB [39], curcumin also restores CK-MB levels in the myocardium through the activation of the CB2 receptor [38].

Troponins are the key regulatory enzymes involved in the contractile mechanism of the heart muscles. Due to the high cardio-specificity of troponin I and T, they are primarily used as markers to diagnose myocardial injury [171], especially in the case of non-ST segment elevation MI [172]. Since troponin I is present exclusively in the myocardial muscles, it serves as a highly specific marker of myocardial injury. In mice with ischemia–reperfusion injury to the myocardium, treatment with the selective CB2 receptor agonist, JWH133, lowers the levels of troponin I in serum, which is attributed to the decreased infarct size [118].

## 8. Conclusions and Future Perspective

The modulation of CB2 receptors can activate and attenuate the different molecular pathways in cardiomyocytes during MI. The presence of CB2 receptors in cardiomyocytes protects them from ischemic injury. While ROS is produced as a result of oxidative stress and cardiac ischemia, the CB2 receptor reduces ROS generation. The CB2 receptor regulates the balance between mitochondrial-mediated apoptosis via the apoptotic protein, as well as autophagy. CB2 receptor activation protects tissues from hypoxia through the iNOS/eNOS cardiac balance to induce NO-triggered vasodilation. Myocardial inflammation is reduced by the CB2 receptor-mediated inhibition of PARP-1 activation. By decreasing pro-inflammatory cytokines, like TNF-α, IL-1β, and IL-6, the CB2 receptor attenuates inflammation, and offers protection against inflammatory cardiac injury. Activation of the CB2 receptor decreases MDA, and increases GSH, catalase, and SOD levels in the ischemic myocardium, to protect tissues from oxidative stress. The CB2 receptor prevents MPTP opening, which reduces ATP depletion and cell death. Pretreatment with a CB2 receptor agonist balances the hemodynamic parameters, as well as the levels of cardiac injury markers, like CK-MB, LDH, and troponin, in the experimental model of MI, which suggests a cardioprotective role of the CB2 receptor. In addition to the myocardium, the CB2 receptor in vascular endothelial cells also plays a protective role against the development of MI. CB2 receptor activation reduces TNF-α-induced production of VCAM-1 and ICAM-1, indirectly showing a protective role in cardiovascular complications.

Taken together, strategies directed at the activation or upregulation of CB2 receptors can produce beneficial effects in ischemic cardiac diseases, including MI. Further, additional investigations are warranted to reveal some unexplored possible underlying molecular mechanisms that might be triggered by the CB2 receptor. For instance, although PPARα plays a pivotal role in protecting the heart during MI, its direct association with the CB2 receptor is not reported. Therefore, the possibility of CB2 receptor-mediated stimulation of PPARα, in addition to PPARγ activation, needs to be explored in order to establish its interaction in MI.

## Data Availability

Not applicable.
